# Localising occult prostate cancer metastasis with advanced imaging techniques (LOCATE trial): a prospective cohort, observational diagnostic accuracy trial investigating whole–body magnetic resonance imaging in radio-recurrent prostate cancer

**DOI:** 10.1186/s12880-019-0380-y

**Published:** 2019-11-15

**Authors:** Sola Adeleke, Arash Latifoltojar, Harbir Sidhu, Myria Galazi, Taimur T. Shah, Joey Clemente, Reena Davda, Heather Ann Payne, Manil D. Chouhan, Maria Lioumi, Sue Chua, Alex Freeman, Manuel Rodriguez-Justo, Anthony Coolen, Sachin Vadgama, Steve Morris, Gary J. Cook, Jamshed Bomanji, Manit Arya, Simon Chowdhury, Simon Wan, Athar Haroon, Tony Ng, Hashim Uddin Ahmed, Shonit Punwani

**Affiliations:** 1 Centre for Medical Imaging, University College London, 2nd floor Charles Bell house, 43-45 Foley Street, London, W1W 7TS UK; 2 Department of Radiology, University College London Hospital, London, 235 Euston Road, London, NW1 2BU UK; 3 Molecular Oncology Group, University College London, Cancer Institute, Paul O’Gorman Building, 72 Huntley Street, London, WC1E 6DD UK; 4 Division of Surgery and Interventional Science, University College London, 4th floor, 21 University Street, London, WC1E UK; 5 Division of Surgery, Department of Surgery and Cancer, Faculty of Medicine, Imperial College London, London, UK; 6 Department of Urology, Charing Cross Hospital, Imperial College Healthcare NHS Trust, London, UK; 7 Oncology Department, University College London Hospital, 235 Euston Road, London, NW1 2BU UK; 8 Comprehensive Cancer Imaging Centre (CCIC), King’s College, London, New Hunt’s House, Guy’s Campus, London, SE1 1UL UK; 9 Department of Nuclear Medicine, The Royal Marsden Hospital NHS Foundation Trust, Down’s Road, Sutton, SM2 5PT UK; 10 Histopathology Department, University College London Hospital, 4th Floor, Rockefeller Building University Street, London, WC1 6DE UK; 11 Institute for Mathematical and Molecular Biomedicine, King’s College London, Hodgkin Building, Guy’s Campus, London, SE1 1UL UK; 12 Department of Applied Health Research, University College London, 1-19 Torrington Place, Fitzrovia, London, WC1E 7HB UK; 13 Department of Cancer Imaging, School of Biomedical Engineering and Imaging Sciences, King’s College London, 4th Floor, Lambeth Wing St. Thomas’ Hospital, London, SE1 7EH UK; 14 Institute of Nuclear Medicine, University College London Hospital, 5th Floor Tower, 235 Euston Road, London, NW1 2BU UK; 15 Urology Department, University College Hospital, Westmoreland Street, 16-18 Westmoreland Street, London, W1G 8PH UK; 16 Oncology Department, Guy’s and St. Thomas’ Hospital, Westminster Bridge road, Lambeth, London, SE1 7EH UK; 17 Department of Nuclear Medicine, St Bartholomew’s Hospital, West Smithfield, London, EC1A 7BE UK; 18 Urology Department, Imperial College Healthcare NHS Trust, London, W2 1NY UK

**Keywords:** Magnetic resonance imaging, Prostate cancer, Radiotherapy, Brachytherapy, Recurrence, Positron emission tomography, Cost comparison, Cost-effectiveness, Economic evaluation

## Abstract

**Background:**

Accurate whole-body staging following biochemical relapse in prostate cancer is vital in determining the optimum disease management. Current imaging guidelines recommend various imaging platforms such as computed tomography (CT), Technetium 99 m (^99m^Tc) bone scan and ^18^F-choline and recently ^68^Ga-PSMA positron emission tomography (PET) for the evaluation of the extent of disease. Such approach requires multiple hospital attendances and can be time and resource intensive. Recently, whole-body magnetic resonance imaging (WB-MRI) has been used in a single visit scanning session for several malignancies, including prostate cancer, with promising results, providing similar accuracy compared to the combined conventional imaging techniques. The LOCATE trial aims to investigate the application of WB-MRI for re-staging of patients with biochemical relapse (BCR) following external beam radiotherapy and brachytherapy in patients with prostate cancer.

**Methods/design:**

The LOCATE trial is a prospective cohort, multi-centre, non-randomised, diagnostic accuracy study comparing WB-MRI and conventional imaging. Eligible patients will undergo WB-MRI in addition to conventional imaging investigations at the time of BCR and will be asked to attend a second WB-MRI exam, 12-months following the initial scan. WB-MRI results will be compared to an enhanced reference standard comprising all the initial, follow-up imaging and non-imaging investigations. The diagnostic performance (sensitivity and specificity analysis) of WB-MRI for re-staging of BCR will be investigated against the enhanced reference standard on a per-patient basis. An economic analysis of WB-MRI compared to conventional imaging pathways will be performed to inform the cost-effectiveness of the WB-MRI imaging pathway. Additionally, an exploratory sub-study will be performed on blood samples and exosome-derived human epidermal growth factor receptor (HER) dimer measurements will be taken to investigate its significance in this cohort.

**Discussion:**

The LOCATE trial will compare WB-MRI versus the conventional imaging pathway including its cost-effectiveness, therefore informing the most accurate and efficient imaging pathway.

**Trial registration:**

LOCATE trial was registered on ClinicalTrial.gov on 18th of October 2016 with registration reference number NCT02935816.

## Background

Prostate cancer (PCa) is the leading male cancer in the United Kingdom and a principal cause of cancer-related mortality with around 46,700 cases diagnosed in 2014 [1]. The incidence of PCa has increased markedly over the past 20 years and it is projected to rise by 12% between 2014 and 2035 to 233 per 100,000 males [1]. Common treatments options for localised prostate cancer are external beam radiation therapy (EBRT), brachytherapy (BRT) or radical prostatectomy [2].

However, biochemical recurrence (BCR) following radiotherapy occurs in 25% of treated men within 5 years, manifesting as a rising prostate-specific-antigen (PSA) [3]. This is strictly a biochemical diagnosis, most commonly defined as an increase in serum PSA of 2.0 ng/ mL above the nadir [4]. Once a PSA relapse has been diagnosed, it is important to determine whether the recurrence has developed at local or distant sites and hence BCR following therapy often initiates an imaging-based assessment of local and metastatic disease in patients.

Local recurrence of PCa is investigated by prostate multi-parametric magnetic resonance imaging (mp-MRI) [5] whilst metastatic disease is assessed by multi-modality imaging. The standard workup to detect PCa metastases usually includes ^99m^Tc bone scan and chest/abdomen/pelvis (CT-CAP) scan (or where available ^18^F-choline or ^68^Ga-PSMA PET-CT) [6].

The workup to exclude distant metastasis is of greatest significance as it is estimated that about half of those who develop radio-recurrent disease have distant metastases (either overt on imaging or micro-metastases) [7]. It is also believed that up to 50% of men who are considered to be free of metastases by conventional staging methods have micro-metastatic disease [8]. This is reflected in prostate cancer recurrence following EBRT or BRT, where biochemical relapse commonly precedes clinical detection of metastases by an average of 7–8 years [9].

Earlier detection of metastatic disease could be the key to better patient stratification and well-informed patient management either by opting for active surveillance or local salvage for low risk individuals or offering systemic therapy at an earlier time point to a high-risk population [10].

Recent technological advances have enabled the reliable whole-body MRI (WB-MRI) staging of cancers within a reasonable scanning time (less than 1 h) [11–14]. There is a clear need for an imaging modality that is capable of improved sensitivity for metastatic disease detection than current conventional imaging [15–17]. Previous meta-analysis has reported the sensitivity of ^99m^Tc bone scan to be less than 50% [18]. Similarly, studies have shown the sensitivity of ^18^F-choline PET to be as low as 50% for the detection of nodal disease when PSA is < 5 ng/ml [19]. Furthermore, CT Scan sensitivity for nodal disease detection in prostate cancer is reported to be much lower with a meta-analysis reporting a sensitivity of 30% [20].

The LOCATE study investigates the diagnostic performance of WB-MRI for detection of nodal and metastatic disease compared to current standard multi-modality imaging comprising ^18^F-choline PET-CT and ^99m^Tc bone scan (+/− CT-CAP) in patients with the radio-recurrent PCa.

### Study objectives

#### Primary objective

To compare the diagnostic accuracy of WB-MRI for regional lymph node and distant metastatic disease detection against an enhanced reference standard of ^18^F-choline PET-CT (or CT CAP) and ^99m^Tc bone-scan for men presenting with BCR following EBRT or BRT.

#### Secondary objectives

1. Inter-observer agreement of WB-MRI for regional lymph node and distant metastatic disease detection
2. Derivation and evaluation of apparent diffusion coefficient (ADC) and fat fraction (FF) signal heterogeneity indices of metastatic disease as predictors of treatment response to ADT
3. Exploration of the significance of the Human Epidermal growth factor Receptor (HER) activated dimer in metastatic castration-resistant prostate cancer
4. Cost-effectiveness of WB-MRI for radio-recurrent prostate cancer staging compared with conventional imaging modalities

## Methods/design

### Study design

**Fig. 1:**
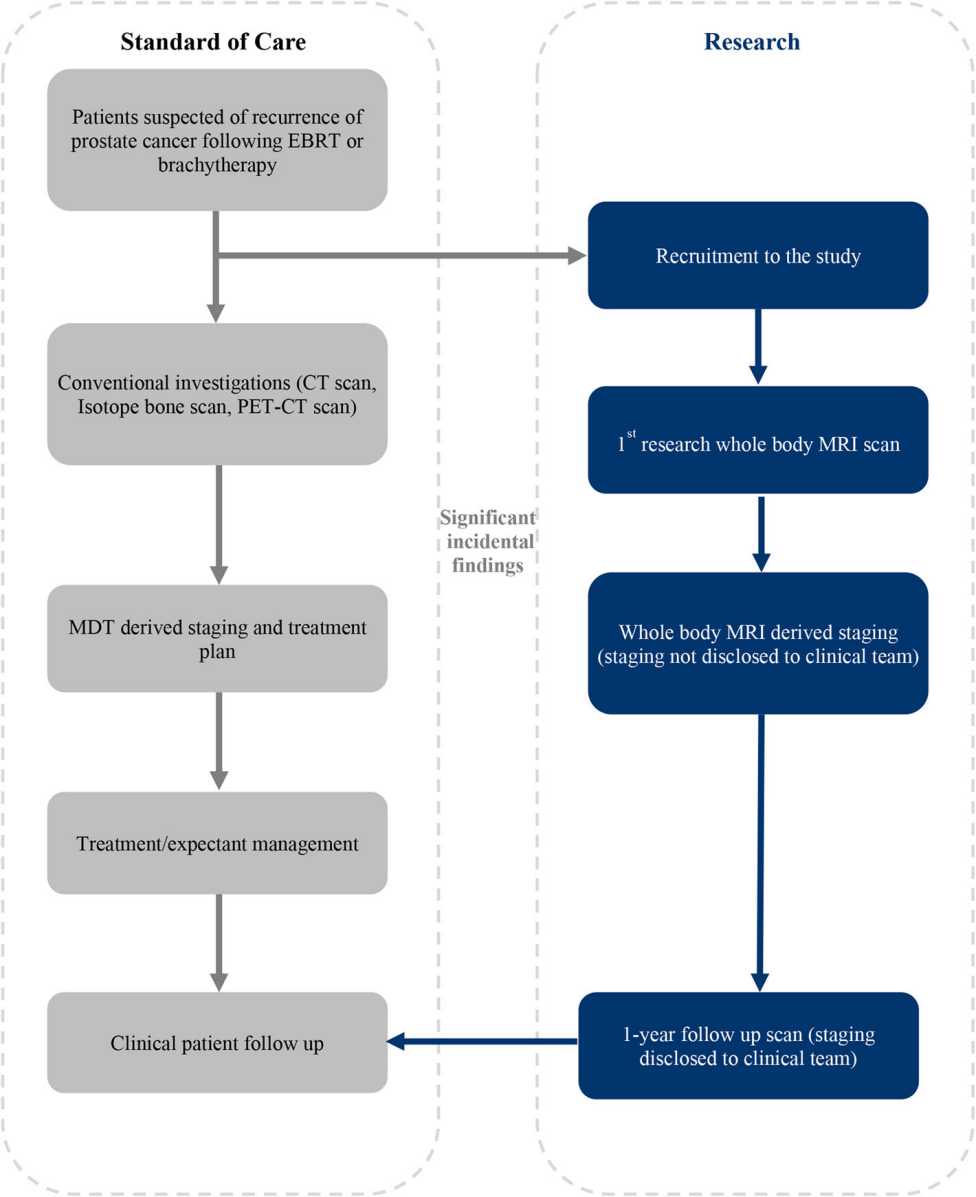
Trial flowchart. The trial flowchart describing the standard of care pathway through which patients were reviewed and clinical decisions made. A parallel research pathway was designed to mimic the standard pathway without interfering in patient care except when clinically significant incidental findings are identified

The comparative diagnostic accuracy study will conform to the Standards for Reporting of Diagnostic Accuracy (STARD) statement [21]. The study is a prospective cohort multi-centre diagnostic study where all participants will have WB-MRI and conventional imaging tests (^18^F-choline PET or CT-CAP and ^99m^Tc bone scan) with findings of both validated against an enhanced outcome-based reference standard. The patient recruitment flowchart is illustrated in Fig. 1.

### Sample size

Based on 50% prevalence of metastases, 130 patients will be required to demonstrate a minimum of a 20% difference in sensitivity for detection of metastases on a per-patient level between WB-MRI and combination of ^18^F-choline PET-CT and ^99m^Tc bone scan (power of 90% and statistical significance cut-off of 0.05).

### Ethical approval

The LOCATE trial received UK Research Ethic Committee (REC) approval from the National research ethics service (NRES) Committee London-Chelsea with REC reference 15/LO/0776 and will be conducted in accordance with the principles of ICH guidelines on good clinical practice in clinical trials and the Research Governance Framework for Health and Social Care (England).

### Recruitment

#### Inclusion criteria

Men who have undergone previous EBRT or BRT with or without neo-adjuvant/adjuvant hormone therapy.

Biochemical relapse as determined by increase in PSA.

#### Exclusion criteria

Men unable to have MRI scan, or in whom artefact would significantly reduce quality of MRI.

Men unable to give informed consent.

#### Informed consent

In all cases, patient information sheets and sample consent forms will be made available to patients a minimum of 24 h prior to the consent procedure. Consent will be obtained at the time of the appointment and prior to commencement of any trial intervention.

### Trial interventions

#### Whole body MRI

Recruited patients will have WB-MRI scan on presentation with BCR and then at 12 months (follow-up). All WB-MRI scans will be performed at University College London Hospital NHS foundation trust (UCLH) imaging department on a 3.0 T scanner (Ingenia; Phillips Healthcare, Best, Netherlands), using a fixed protocol (Table 1). For participants’ comfort, the WB-MRI protocol will be limited to a maximum of 1-h scan time.

**Table 1:** Whole Body MRI sequence parameters

	T2-TSE	mDixon (pre- and post-contrast)	DWI (b0, 1000)
Slice orientation	Transverse	Coronal	Transverse
Echo time (ms)	80	2.303	17
Repetition time (ms)	1214.69	3.5	6304.5
Space between slices	5.5	2.5	5.5
Number of slices	40	120	40
Slice thickness (mm)	5	5	5
Acquisition matrix	500*497	240*238	124*118
Echo train length	89	2	39
Number of averages	1	1	2
Pixel bandwidth (Hz)	538	1847	3354
Pixel spacing	0.78/0.78	1.04/1.04	2.08
Flip angle	90	15	90

*TSE* Turbo spin echo, *DWI* Diffusion weighted imaging

#### Conventional imaging

Recruited patients will have the standard imaging investigations according to local and national guidelines as below:

#### Multi-parametric prostate MRI

A standard prostate mp-MRI protocol as defined by the UK consensus guidelines on prostate MRI [22] will be used to locally stage prostate cancer. Conventional T1 and T2-weighted images of the prostate will be supplemented with diffusion weighted imaging (DWI) MRI (as per hospital site protocol); and +/− dynamic contrast enhanced (DCE) T1-weighted images.

#### Computed tomography

CT scans of the chest/abdomen/pelvis will be performed with intravenous contrast agent using the local standard protocols.

#### ^99m^Technetium bone scan

Bone scans will be performed using ^99m^Tc labelled diphosphonates administered through intravenous injection. For prostate cancer patients with suspected bone metastases, the standard protocol employed at the hospital site will be used. As a guide, whole body imaging will be conventionally performed with anterior and posterior views, 256 × 1024 matrix and energy window(s) of 140 KeV.

#### Positron emission tomography-computed tomography (PET-CT)

^18^F-choline PET-CT will be acquired using a dedicated combined PET/64-detector-CT (VCT-XT Discovery, GE-Healthcare Technology, Chicago, Illinois), CT will be performed (for attenuation correction) using 64 × 3.75 mm detectors, a pitch of 1.5 and a 5 mm collimation (120 kVp and 10 mA in 0.8 s). Maintaining the patient position, a whole-body choline PET emission scan will be performed and cover an area identical to that covered by CT [23].

### Non-imaging trial interventions

#### Blood samples

Blood samples will be taken from recruited patients, by an appropriately trained member of the trial team, at the time of each WB-MRI (staging and then at 12-month follow-up). Blood samples will be used for exosome and phenotypic [Fluorescence-activated cell sorting (FACS) analysis] / DNA-RNA analysis. In total a maximum of 30 ml of blood will be taken:

#### Up to 3 acid citrate dextrose solution (ACD) tubes: to collect ~ 15 ml blood for serum separation

Up to 3 ethylenediaminetetraacetic acid (EDTA) containing tubes for peripheral blood mononuclear cells (PBMC) isolation and further FACS analysis, functional assays, and DNA/RNA isolation.

Frozen aliquots-samples will be transported by courier service in dry ice-containing carrier boxes within 24 h after obtaining the blood sample. Samples will be processed at University College London (UCL) and King’s College London (KCL) laboratories:

UCL: Exosome purification and analysis. Protein-DNA-RNA isolation and analysis.

KCL: PBMC’s isolation, FACS analysis and functional assays.

### Health economic data collection

Data collection for the health economic analysis will be primarily concerned with cost data associated with the diagnosis and treatment of metastatic prostate cancer. Routinely collected administrative data from UCLH will be used to determine the relevant costs associated with the diagnostics test. To determine the consequence of detected metastatic disease on the treatment pathway, clinical experts will be interviewed, and patient notes will be reviewed noting changes to medication, radiotherapy, surgery, outpatient visits, inpatient visits and day cases, where possible. Data from existing trials such as STAMPEDE [24] and FORECAST [7] will be used to inform other economic parameters such as the health-related quality of life as these are not being collected in LOCATE.

### Reporting of trial imaging

#### Initial WB-MRI scan

The study will be reported by two experienced radiologists, blinded to all other imaging investigations, initially independently and then in consensus using a locked sequential read paradigm (LSR). The images will be reviewed for the presence of nodal (N) and metastatic (M) disease and each radiologist will record the presence/absence of disease on the study-specific proforma.

The initial WB-MRI read results will not be revealed to the clinical team. Where a significant additional/incidental finding is demonstrated this will be reported as per standard clinical practice and relayed to the clinical team.

A 1–6 Likert scale [23, 25] will be used for scoring as has been previously described for WB-MRI studies. 1 – disease definitely not present, 2 – probably not present, 3 – possibly not present, 4 – disease possibly present, 5 – probably present and 6 – definitely present.

Scoring will be conducted based on evaluation of imaging features of lymph nodes and bone metastases as indicated below in Table 2 .

**Table 2:** Features suggestive of adverse and benign changes in both nodes and bones

	Benign features	Adverse features
Node	Lymph node <5 mm in short axis diameter (SAD) with no concerning features on DWI, T2 weighted or contrast enhanced imaging and definite benign features e.g. (i) Fatty hilum, (ii) Oval nodal morphology with clearly defined and (iii) Regular nodal border and contours.	Concerning features could be described as (i) Size above normal limit e.g. > 10 mm for most, (ii) Loss of fatty hilum, (iii) Irregular border, (iv) Asymmetric high DWI signal intensity, (v) Low T2 signal
Bone	Normal appearing bone with uniform moderate-high signal on T1 weighted imaging and low-moderate signal on T2 weighted imaging. Focal lesions demonstrating clearly incidental/benign multiparametric signal characteristics and/or location	(i) Increase in DWI high b-value signal vs. background noise. (ii) Low signal intensity on T1 and (iii) Intermediate to high signal intensity on T2 (iv) Lesional contrast enhancement

*DWI* Diffusion weighted imaging

#### 12-month follow-up WB-MRI

The study will be reported by the same pair of radiologists who reported the baseline scans. The findings on the follow-up study will be compared with the baseline WB-MRI with access to all conventional imaging. The presence of changes in size of lesions evident on the baseline WB-MRI and/or resolution of lesions evident on baseline WB-MRI will be recorded. Any discrepant findings will be reviewed, and the status of these lesions will be determined in a consensus panel review.

### Derivation of enhanced reference standard

**Fig. 2:**
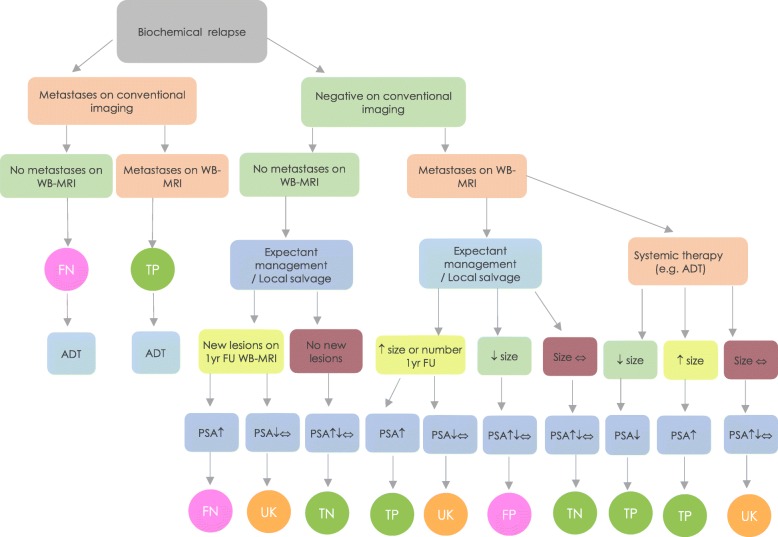
Enhanced reference standard derivation flowchart. FN: False Negative, TP: True Positive, FP: False Positive, TN: True Negative, UK: Unknown, ADT: Androgen Deprivation Therapy

Figure 2 illustrates the derivation of a per-patient level enhanced reference standard (ERS).

Patients will be divided into those with and without metastases evident on conventional imaging. For those with metastases (left arm of Fig. 2), concordant positive WB-MRI findings will be deemed to be true positive and discordant WB-MRI findings will be considered to be false negative.

For patients without metastases on conventional imaging (right arm of Fig. 2), clinical and biochemical follow-up together with changes between baseline and follow-up WB-MRI will be used to enhance the reference standard.

Where baseline WB-MRI was negative and concordant with conventional imaging, the presence of new lesions (at follow-up WB-MRI ± repeat routine imaging) coupled with a PSA rise over the 12-month will classify the baseline WB-MRI as false negative, i.e. disease was present but not visualised. Whereas the absence of new lesions regardless of PSA status over the 12-month follow-up period will confer baseline WB-MRI true negative status.

For positive findings on baseline WB-MRI discordant with conventional imaging, the baseline WB-MRI will be deemed true positive when a new lesion declared itself (on follow-up WB-MRI ± routine imaging) or the visualised lesion on baseline WB-MRI enlarged (on follow-up WB-MRI); and this was accompanied with a PSA rise within the 12-month follow-up period. Whereas, the baseline WB-MRI will be deemed false positive when the lesion evident at WB-MRI resolved or reduced in size at follow-up (in the absence of systemic therapy) regardless of PSA change. Lesions that resolved following systemic therapy (which is initiated in the 1-year follow-up period irrespective of negative conventional baseline imaging) with corresponding decrease in PSA will be classified as true positive.

Finally, patients with negative baseline conventional imaging with discordant lesion and PSA changes over the 12-month follow-up (i.e. lesion size/number increase without corresponding PSA increase) will be classified as unknown.

The section below describes the thresholds for PSA and lesion size changes, and they will be interpreted in the context of treatment intervention and subsequent treatment response or progression.

PSA progression defined as ≥25% and ≥ 2 ng/mL increase in PSA recorded at least 12 weeks from baseline or start of treatment and confirmed on a 2nd reading 3–4 weeks later as previously defined [26, 27].

PSA response defined as ≥50% decline from baseline or start of treatment and confirmed on a 2nd reading 3–4 weeks later [28, 29].

Nodal size change Increase of ≥2 mm and decrease of ≥3 mm in short axis diameter on contrast enhanced mDixon sequence will be considered true change (progression and response respectively) rather than a measurement variation, with a 95% confidence interval. (Based on unpublished data from repeatability studies carried out as part of the LOCATE trial).

Bone lesion (disease progression) ≥ 25% increase in size (sum of short and long axes diameters) of lesions on CT, or MRI [30, 31].

Bone lesion (treatment response) ≥ 50% decrease in size (sum of short and long axes diameters) of lesions on CT/ MRI or normalisation of signal intensity on MRI [30, 31].

Soft tissue lesion size change (disease progression) At least a 20% increase in size of lesion taking as reference the baseline diameter of the lesion [32].

Soft tissue lesion size change (treatment response) at least a 30% decrease in the size of lesion, taking as reference the baseline diameter or disappearance of the lesion [32].

### Image analysis

#### Reporting of conventional imaging

The conventional imaging (^18^F-choline PET-CT, CT-CAP and ^99m^Tc Bone Scan) will be independently reported by two radiologists and nuclear medicine physicians, later reviewed in consensus to clarify any discrepant findings. The report will be transferred to a proforma and final nodal (N) and metastatic (M) stages will be derived base on the reported proforma.

#### Comparison of conventional imaging with WB-MRI

The research team will correlate the conventional imaging proforma with the baseline WB-MRI proforma and follow-up WB-MRI findings. The radiologists and nuclear medicine physicians in consensus will review all discrepancies, between conventional and WB-MRI scans. Anatomical matching errors resulting from discrepancies in ascribing disease location will be recorded and corrected [33]. Residual discrepancies will be highlighted, categorised as perceptual and technical and statistically assessed [33]. The per-patient sensitivity and specificity of WB-MRI will be determined against the enhanced reference standard before and after correction for any anatomical matching discrepancies and perceptual errors.

Patients who are unable to attend the 1 year follow up WB-MRI may not be included in the diagnostic accuracy analysis. Efforts will be made to minimise amount of missing data. A statistician’s advice will be sought especially on how to manage ‘missing not at random’ data (if any).

### Sub-study analysis

#### The significance of the human epidermal growth factor receptor (HER) activated dimer

The blood sample will be used to investigate the significance of the Human Epidermal growth factor Receptor (HER) activated dimer in recurrent prostate cancer. Up-regulation of the HER1 (EGFR)/HER3 dimer was recently found, for the first time, to limit the efficacy of anti- epidermal growth factor receptor (EGFR) treatment in human breast cancer, as directly shown by imaging of residual disease; suggesting the potential of combined EGFR/HER3-targeted treatment [34]. This molecular signalling rewiring is hypothesised to constitute a pathway of resistance to hormonal treatment in prostate cancer as well. We intend to quantify the EGFR/HER3 heterodimer using fluorescence lifetime imaging microscopy (FLIM) in formalin-fixed prostate cancer tissues as well as in matched circulating exosomes from patient-derived plasma. We will then correlate the heterodimer quantification with tumour genomic changes such as phosphatase and tensin homolog (PTEN) mutation/deletion. The patient-derived correlative experiments will be complemented by mechanistic in vitro experiments that investigate the effect of EGFR and phosphatidylinositol 3-kinase (PI3K)/AKT inhibitors on HER dimer formation in castration-resistant and sensitive prostate cancer cells, and their exosomes released into the culture supernatants. In the clinical setting, the tissue and exosome-derived HER dimer measurements will be combined with WB-MRI findings to assess their value and translation as predictive biomarkers of clinical outcome and response to treatment.

#### Heterogeneity (across metastatic sites) of WB-MRI signals for prediction of ADT response

For patients undergoing ADT treatment, retrospective quantitative analysis of WB-MRI’s ADC and FF signal intensities will be performed to derive heterogeneity indices across confirmed true positive metastatic sites. The heterogeneity indices will inform Bayesian models to predict high-risk patients with aggressive disease that progress on ADT within 12-months of treatment.

### Health economic analysis

The health economic analysis will compare the incremental costs and effects of WB-MRI for metastatic disease detection compared with conventional imaging from a national health service (NHS) perspective and thus determine whether it is an efficient use of NHS resources. From an economic viewpoint, the cost-effectiveness of WB-MRI versus conventional imaging is determined by two main stages of the pathway: the treatment decision pathway and the treatment pathway (and its associated disease pathway). The former includes the time from presentation to treatment decision by the clinician and includes the imaging tests received; the latter includes the time period following the treatment decision and subsequent disease progression. In patients for whom the treatment decision with WB-MRI is the same as that with conventional staging, the subsequent disease pathways will be the same. Where the treatment decision with WB-MRI is different, the disease pathway will be different, yielding potentially different costs and health outcomes (and hence the patient’s quality of life).

The precise nature of the economic analysis will therefore depend on the degree of concordance between treatment decisions provoked by WB-MRI versus conventional imaging.

#### Scenario 1

There is concordance between the treatment decisions associated with WB-MRI and conventional imaging. In this case, the cost components included in the analysis will be conventional metastases imaging tests (as described above) and the costs of treating adverse events associated with conventional staging. The volume of resource use and unit costs will be taken from standard published sources, hospital administrative datasets and from trial data. Since the two algorithms yield the same treatment decisions, cost-effectiveness depends on the incremental cost only of WB-MRI versus conventional imaging.

#### Scenario 2

There is discordance between the treatment decisions associated with WB-MRI and standard imaging. In this case, cost-effectiveness depends on the incremental cost and effects of the treatment decision pathway and the subsequent disease pathway associated with WB-MRI versus conventional staging.

The economic evaluation will also take into consideration the accuracy of WB-MRI versus conventional standard imaging techniques. Specifically, we will model the consequences, in both costs and effects, of true positives, false positives, true negatives and false negative outcomes. Thus, the economic study will evaluate both clinical and economic consequences of the novel imaging technique.

Cost-effectiveness will be calculated as the mean cost difference between WB-MRI versus conventional imaging divided by the mean difference in outcomes (as measured quality-adjusted life years; QALYs) to give the incremental cost-effectiveness ratio (ICER). Also, a cost-consequence analysis will be performed by calculating the mean incremental costs per incremental gain (or loss) in clinical natural units, for example cost per metastases identified.

Finally, to address uncertainty in our clinical and economic parameters, we will perform a comprehensive set of sensitivity analyses on our model estimates. Probabilistic sensitivity analysis using non-parametric bootstrapping methods will be performed. Outputs from this will inform a cost-effectiveness acceptability curve, which shows the probability that WB-MRI is cost-effective at different values of the NHS’ willingness to pay for an additional QALY. We will also subject the economic model to extensive one-way sensitivity analysis to identify key parameters that drive cost-effectiveness.

## Discussion

At the time of writing, the LOCATE trial is the largest prospective multi-centre trial to compare WB-MRI with conventional imaging pathway specifically in patients with radio-recurrent prostate cancer.

The LOCATE trial will be informative because it compares comprehensive imaging pathways and takes into consideration the cost-effectiveness of each pathway. This trial will therefore provide valuable information to guide potential implementation of new imaging platforms for prostate cancer management pathways.

The results from the LOCATE trial would be relevant to the current clinical management pathway, specifically, should WB-MRI be advocated in the NHS in the setting of BCR.

The LOCATE trial design includes important features: unlike most other trials, the initial finding on WB-MRI will be validated using 1 year follow up WB-MRI scans, corroborating the findings on initial scan in the context of patient management. In this way, we minimise the potential overcalling of disease positivity by WB-MRI that could lead to an overestimated sensitivity of WB-MRI compared to conventional imaging techniques.

Additionally, and in order to define the most cost-effective imaging pathway for the health care system, we will perform an economic evaluation which includes a comprehensive set of sensitivity analyses to identify uncertainty in both clinical and economic estimates. Whilst recent trial designs incorporating WB-MRI are including health economic analysis [35–38], such information is still unavailable for prostate cancer imaging pathways.

The LOCATE study has some potential limitations. Firstly, we are using a combined biochemical/imaging follow-up to derive an enhanced reference standard against which the results of WB-MRI will be analysed. Although histopathological confirmation would have been preferable in cases where a discrepancy exists between WB-MRI and conventional imaging tests, such approach is technically challenging and ethically not feasible [39].

Secondly, whilst the qualitative and metastatic staging findings can be generalised to other imaging platforms, such generalisability would be limited in quantitative biomarker imaging analyses (heterogeneity index). However, ongoing work is being carried out to improve standardisation of quantitative MRI imaging across different platforms that could ultimately aid in generalisation of such technique [39, 40].

Finally, it is important to note that ^68^Ga-PSMA PET-CT within a short time has led to significant advances in prostate cancer imaging both in primary and recurrent settings [41, 42]. However, there is yet to be large prospective multi-centre studies to assess its diagnostic accuracy in comparison to WB-MRI, and the start of the LOCATE trial predated availability of ^68^Ga PSMA PET-CT at our institution and therefore a head to head comparison was not possible. More importantly, there is significant cost difference between the two modalities and PSMA is routinely not available in many centres in the UK.

### Study status

At the time of submission, the study is currently at the data collection stage and full trial data analysis is expected to begin shortly.

## Data Availability

Not applicable.

## Abbreviations

ACD: Acid citrate dextrose
ADC: Apparent diffusion coefficient
ADT: Androgen deprivation therapy
BCR: Biochemical relapse
BRT: Brachytherapy
CT: Computed tomography
DWI: Diffusion weighted imaging,
EBRT: External beam radiotherapy
EDTA: Ethylenediaminetetraacetic acid
EFGR: Epidermal growth factor receptor
EPI: Echo planar imaging
FACS: Fluorescence-activated cell sorting
FF: Fat fraction
FLIM: Fluorescence lifetime imaging microscopy
HER: Human epidermal growth factor receptor
ICER: Incremental cost-effectiveness ratio
MDT: Multi-disciplinary team
MRI: Magnetic resonance imaging
NHS: National health service
PACS: picture and archiving system
PBMC: Peripheral blood mononuclear cell
PCa: Prostate cancer
PET: Positron emission tomography
PI3K/AKT: Phosphatidylinositol 3-kinase
PSA: Prostate specific antigen
QALY: Quality-adjusted life year
TSE: Turbo spin echo
WB-MRI: Whole-body magnetic resonance imaging
